# Caspase-3/GSDME dependent pyroptosis contributes to offspring lung injury induced by gestational PFOS exposure via PERK/ATF4 signaling

**DOI:** 10.1007/s00204-023-03626-w

**Published:** 2023-11-13

**Authors:** Cong Li, Huishan Zhang, Jiali Mo, Jingye Zuo, Leping Ye

**Affiliations:** 1 Department of Pediatrics, Peking University First Hospital, No.1 Xi’an Men Street, West District, Beijing, 100034 China; 2 Department of Respiratory Medicine, Shanghai Children’s Medical Center, School of Medicine, Shanghai Jiao Tong University, Shanghai, 200120 China

**Keywords:** Perfluorooctane sulfonate, Pyroptosis, GSDME, PERK/ATF4 pathway, Lung injury

## Abstract

Perfluorooctane sulfonate (PFOS) is widely used in industry and consumer products. Previous studies have showed that PFOS gestational exposure is associated with offspring lung damage in rat. However, the underlying mechanisms remain poorly understood. In this study, we investigated the role of gasdermin E (GSDME) in lung injury of offspring and its underlying mechanisms using in vivo and in vitro approaches. Pregnant SD rats were exposed to PFOS (1 mg/kg BW/d) between gestational day 12–18, and the lung tissue of the offspring was evaluated on postnatal day 7. PFOS treated animals exhibited alveolar septal thickening and inflammation-related damages, with an increased expression of GSDME in alveolar type II epithelial cells (AECII). Furthermore, in vitro experiments demonstrated that PFOS exposure (with 225 μM and up) upregulated the caspase-3/GSDME signaling pathway in AECII. Also, ultrastructure analysis revealed significant changes in the endoplasmic reticulum (ER) structure in PFOS-induced pyroptotic cells, which is consistent with the ER stress detected in these cells. Additionally, PFOS exposure led to increased expression of ER stress-related proteins, including p-PERK, p-eIF2α, ATF4, and CHOP. Subsequently, using specific inhibitors, we found that the PERK/ATF4 pathway acted as an upstream signal regulating GSDME-dependent pyroptosis. Overall, our findings show that GSDME-dependent pyroptosis plays a crucial role in the lung injury induced by gestational PFOS exposure, and the PERK/ATF4 pathway may function as a possible mediator of this process.

**Supplementary Information:**

The online version contains supplementary material available at 10.1007/s00204-023-03626-w.

## Introduction

Perfluorooctane sulfonate (PFOS) is a widely utilized perfluoroalkyl compound in both industry and household products. Owing to its broad distribution and stable physical and chemical properities, PFOS can be persistent in nature and infiltrate the human body through multiple routes of exposure and accumulate in various organs (Ahmed et al. 2020; Chu et al. 2022; Guo et al. 2018; Sunderland et al. 2019), such as liver, lungs, and kidneys (Jian et al. 2018; Wang et al. 2018). In addition, PFOS can cross the placental barrier and enter the fetus upon its exposure during pregnancy (Olsen et al. 2007). Prenatal exposure to PFOS is associated with the adverse effects on development of fetal organs (Sevelsted et al. 2022), in particular, the lung tissue (Mamsen et al. 2019). Both acute and chronic exposures to PFOS have been shown to induce the production of inflammatory cytokines and related-damages in lung tissue (Wang et al. 2022b). In human, such exposure is accompanied by an increase in the frequency of lower respiratory tract infections in infants and young children before the age of 10 (Impinen et al. 2018). However, it is still unclear how gestational PFOS exposure may lead to lung injury in offspring.

Pyroptosis, an inflammatory form of cell death, is characterized by cellular swelling and membrane damage, resulting in cell rupture and release of intracellular contents, and this process is mediated by the gasdermin family (Sborgi et al. 2016). Recent studies discovered that gasdermin E (GSDME) is involved in pyroptosis of lung cells induced by multiple factors (Wang et al. 2017). For example, exposures of A549 cells to paclitaxel and cisplatin can activate caspase-3 and GSDME related pyroptosis which is closely associated with mitochondrial damage (Zhang et al. 2019). Likewise, Paraquat has been shown to enhance the production of reactive oxygen species (ROS) in bronchial epithelial cells, leading to the activation of caspase-3 and the triggering of GSDME-dependent pyroptosis and lung injury (Tang et al. 2023). All of these findings implicate the crucial role of GSDME-mediated pyroptosis in lung pathology. Nevertheless, it is still unknown whether the lung injury of offspring rats caused by gestational PFOS exposure relates to GSDME mediated pyroptosis, and further investigations are required to elucidate its specific mechanism.

In this study, we used gestational SD rats and two alveolar type II epithelial cell (AECII) lines (L2/RLE-6TN)as in vivo and in vitro models to examine how prenatal PFOS exposure impacts lung injury in rat offspring, with a focus on understanding the role of PFOS in GSDME-dependent pyroptosis. Our findings demonstrate that PFOS exposure during pregnancy triggers lung injury in offspring rats via caspase-3/GSDME-dependent AECII pyroptosis. Moreover, we have identified that the PFOS-related pyroptosis was mediated by protein kinase RNA-like ER kinase (PERK) and activating transcription factor 4 (ATF4) signaling pathway.

## Materials and methods

### Reagents

PFOS (C8F17KO3S, MW: 538.22, purity ≥ 98%, CAS: 2795-39-3, CAT#: 77282) was procured from Sigma-Aldrich (St. Louis, MO, USA). The caspase-3 inhibitor zDEVD-FMK (CAS: 210344-95-9, CAT#: HY-12466) and PERK inhibitor GSK2606414 (CAS: 1337531-36-8, CAT#: HY-18072) were obtained from MedChemExpress (Monmouth Junction, NJ, USA).

### Animals and treatment

Sprague–Dawley rats (250–300 g body weight) of 9 weeks of age, with specific pathogen-free (SPF) status, were procured from Beijing Vital River Laboratory Animal Technology Co., Ltd. (Beijing, China) and housed in the animal facility of Peking University First Hospital. Female rats were mated with a male rat (female: male, 1:1), and the pregnant rats were maintained at an ambient temperature of 23 ± 2 °C with a 12:12-h light–dark cycle and free access to a standard rat chow and drinking water. Rats were randomly divided into two groups, with eight rats in each group. From gestational day 12 to day 18, the rats were administered different doses of PFOS (0 or 1 mg/kg body weight per day in 0.5% Tween 20) via gavage. The PFOS dose was chosen based on previous studies (Zhang et al. 2021). After delivery, lung tissues were collected on postnatal day 7 (PND7) and the whole middle lobe of the right lung was meticulously excised and fixed in a 4% paraformaldehyde solution for subsequent histological and immunohistochemical analyses. All animal procedures were performed in accordance with the National Institutes of Health Guide for the Care and Use of Laboratory Animals, according to protocols (No. 2023053, Beijing, China) approved by the Peking University First Hospital.

### Cell culture and treatment

The AECII cell lines, L2 and RLE-6TN, were procured from the American Type Culture Collection (ATCC, Manassas, VA, USA) and served as the in vitro models for PFOS exposure. These cells were cultured in Dulbecco's Modified Eagle Medium with High Glucose (DMEM, Gibco, Rockville, MD, USA) supplemented with 10% fetal bovine serum (Sigma-Aldrich, St. Louis, MO, USA). Cells were maintained in a 37 °C incubator with 5% CO2. To knock down GSDME expression, siRNA sequences targeting the GSDME gene were designed as followed 5′-GGAAGGUCAAGCUGAAUGU-3′, 5′-ACAUUCAGCUUGACCUUCC-3′ (Tsingke, China), and was transfected into cells in a six-well plate using Lipofectamine 3000 (Invitrogen, Carlsbad, CA, USA) according to the manufacturer’s instructions. PFOS, GSK2606414 and zDEVD-FMK were individually dissolved in dimethyl sulfoxide (DMSO; Solarbio, Shanghai, China) to prepare a stock solution and was freshly diluted in the culture medium to right before each application. The maximal DMSO in the culture medium was never exceeding 0.2%.

### Cell viability and LDH release assays

RLE-6TN and L2 cells were seeded at a density of 2 × 10^3^ cells per well in 96-well plates. Subsequently, the cells were treated with various concentrations of PFOS (0, 65, 100, 150, 225, 300, and 450 μM) for a duration of 24 h. The Cell Counting Kit-8 (Beyotime, Shanghai, China) was employed to assess cell viability by incubating the cells at 37 °C for 1 h and measuring the optical density at 450 nm. Lactate dehydrogenase (LDH) was determined in the culture supernatant, using the LDH kit (Nanjing Jiancheng Bioengineering Institute, Nanjing, China) according to the manufacturer's instructions.

### Flow cytometry assay of cell death

RLE-6TN and L2 cells were seeded in 10cm Petri dishes at a density of 4 × 10^5^ cells per dish and subsequently exposed to different concentrations of PFOS (0, 100, 150, and 225 μM) for 24 h. Then, cells were detached by trypsin digestion and collected in centrifugation tubes. The dead cells were labeled with Annexin V-FITC/PI detection kit (Dojindo, Japan). Specifically, propidium iodide (PI) and Annexin V were added to the tubes as per the manufacturer's instructions, and the cells were incubated at room temperature in the dark for 15 min. Subsequently, the samples were analyzed using a BD FACSCalibur flow cytometer (BD, Becton, Dickinson and Company, USA).

### Endoplasmic reticulum integrity assessment

Endoplasmic reticulum (ER) Tracker Red (Beyotime, Shanghai, China) was used as a fluorescent probe to evaluate ER swelling. Briefly, RLE-6TN and L2 cells were seeded in 1.5cm confocal petri dishes at a density of 7.5 × 10^4^ cells per dish, cultured overnight and then exposed to different concentrations of PFOS (0, 100, 150, and 225 μM) for 24 h, and subsequently washed twice with Hanks’ Balanced Salt Solution (HBSS) containing Ca^2+^ and Mg^2+^. The cells were treated with a pre-warmed ER Tracker solution (1:2000) at 37 °C for approximately 15 min and were washed twice with cell culture solution. Subsequently, a laser scanning confocal microscope (Nikon Eclipse, Tokyo, Japan) was used to examine and capture images of the treated cells.

### Real-time quantitative PCR (qPCR)

Total RNA was isolated from the cells using TRIzol reagent (Beyotime, Shanghai, China). Subsequently, the RNA was reverse transcribed into complementary DNA using the ReverTra Ace qPCR RT Kit (TOYOBO, Osaka, Japan). qPCR was performed using SYBR Green Real-time PCR Master Mix (TOYOBO, Osaka, Japan), and cDNA was amplified according to the following steps: denaturation at 95 ℃ for 2 min, followed by denaturing/annealing at 95 ℃ (10s) and 60 ℃ (40s) for 40 cycles. Primers used included activating transcription factor 4 (*ATF4*), *ATF6*, X-Box binding protein 1 (*XBP1*), tumor necrosis factor-α (*TNF-α)*, interleukin 8 (*IL8)*, and high mobility group box-1 protein (*HMGB1*) (Table 1). The mRNA expression levels were determined using the 2^−ΔΔCT^ method, with β-actin serving as an internal standard.

**Table 1:** The primers used for RT-PCR detection

Gene	Forward sequence (from 5′ to 3′)	Reverse sequence (from 5′ to 3′)
ATF4	TGGCTATGGATGGGTTGGTC	GCTCATCTGGCATGGTTTCC
ATF6	ATCACCTGCTATTACCAGCTACCAC	TGACCTGACAGTCAATCTGCATC
XBP1	AGGTCTCAGAGGCAGAGTCCAAG	AAGAGGCAACAGCGTCAGAATCC
TNF-α	TAATGCTGATTTGGTGACCAGG	GTAGGGCGATTACAGTCACGG
IL8	GAACATCCAGAGTTTGAAGG	GGTAGAGAACGGATGAACAC
HMGB1	GAAGCCGAGAGGCAAATGTCC	TACCTCTGTAGGCAGCAATAC

### Western blotting

Cells and tissues were lysed in RIPA lysis buffer (Beyotiom Biotech, China), and protein concentration was determined by BCA protein assay kit (Beyotiom Biotech, China). Total proteins were separated for each sample using 10% SDS-PAGE and then transferred to PVDF membrane (Millipore, Burlington, USA). The membrane was blocked in 5% skim milk for 1 h and incubated with the primary antibody at 4 ℃ overnight (Supplementary Table 1). After washing with Tris-buffered saline with 0.1% Tween 20 (TBST) three times, membranes were incubated with secondary antibodies for 1 h at room temperature. The target protein bands were visualized using Super Enhanced chemiluminescence detection reagent (Applygen Technologies Inc., Beijing, China).

### Histopathological and immunohistochemical (IHC) procedures

The lung tissues were fixed with 4% paraformaldehyde and embedded in paraffin, Then, 3 µm-thick sections were stained with hematoxylin–eosin staining (HE) and IHC procedures as described previously (Zhang et al. 2021). Lung pathological changes were evaluated by Smith lung injury scores and the radial alveolar count (RAC) system, following a previously established protocol (Zhang et al. 2021). During the scoring process the thickness of alveolar septa was also quantified. For IHC analysis, paraffin sections of lung tissue were first dewaxed with xylene and gradient alcohol. Then, the antigen was retrieved by microwaving the tissue in citric acid buffer (10 mM, pH 6.0) for 20 min to repair the antigen. Endogenous peroxidase was blocked by incubation with 3% hydrogen peroxide for 10 min at room temperature. Subsequently, antibodies for GSDME (1:50), p-PERK (1:50), p-eIF2a (1:50), ATF4 (1:50) or CHOP (1:50) were incubated respectively at 4℃ overnight. After washing in PBS for 10 min, the membrane was further incubated with goat-anti-rabbit or goat-anti-mouse secondary antibody (1:50) for 20 min at room temperature. The targeted proteins were visualized by diaminobenzidine (ZSGB-BIO, Beijing, China) chromogen, and counterstained by hematoxylin (Solarbio, Beijing, China).

### Immunofluorescence analysis

Antigen retrieval was performed with paraffin sections in citric acid buffer (10 mM, pH 6.0) for 20 min. After nonspecific binding on the section was blocked with 10% goat serum at room temperature for 30 min. The sections were incubated with anti-GSDME and anti-ATF4 primary antibodies (1:200) overnight at 4 ℃, followed by incubations with the corresponding secondary antibodies (1:500 dilution) in the dark at room temperature for 2 h. Finally, the nuclei were visualized using 4′,6-diamidino-2-phenylindole (DAPI, blue). All sections were examined and recorded with fluorescent and/or confocal microscopies.

### Ultrastructure assessment by electron microscopy

About 4 × 10^5^ cells were seeded in 10cm Petri dishes. Following a 24-h treatment with PFOS (0 or 225 μM), cells were freed from the dishes by digesting with 0.25% trypsin/0.02%EDTA, and fixed with 2.5% glutaraldehyde. Ultrathin sections were prepared and stained using saturated uranyl acetate and lead staining solution at room temperature for 15 min. The ultrastructural changes were observed using transmission electron microscopy (TEM).

### Statistical analyses

Statistical analyses were performed using GraphPad Prism 9 software. ImageJ software was utilized for analyzing protein band densities, while FlowJo software was employed for analyzing flow cytometry data. Data were expressed as Mean ± SD of at least 3 animals or 3 biological replicates. Two-sample comparisons were assessed using independent sample t-tests, while multiple group comparisons were performed using one-way ANOVA followed by Tukey’s post-hoc test. Statistical significance was defined as *p < 0.05, **p < 0.01, ***p < 0.001, and ****p < 0.0001, indicating the level of significance.

## Results

### Effects of PFOS on lung morphology and GSDME expression

**Fig. 1:**
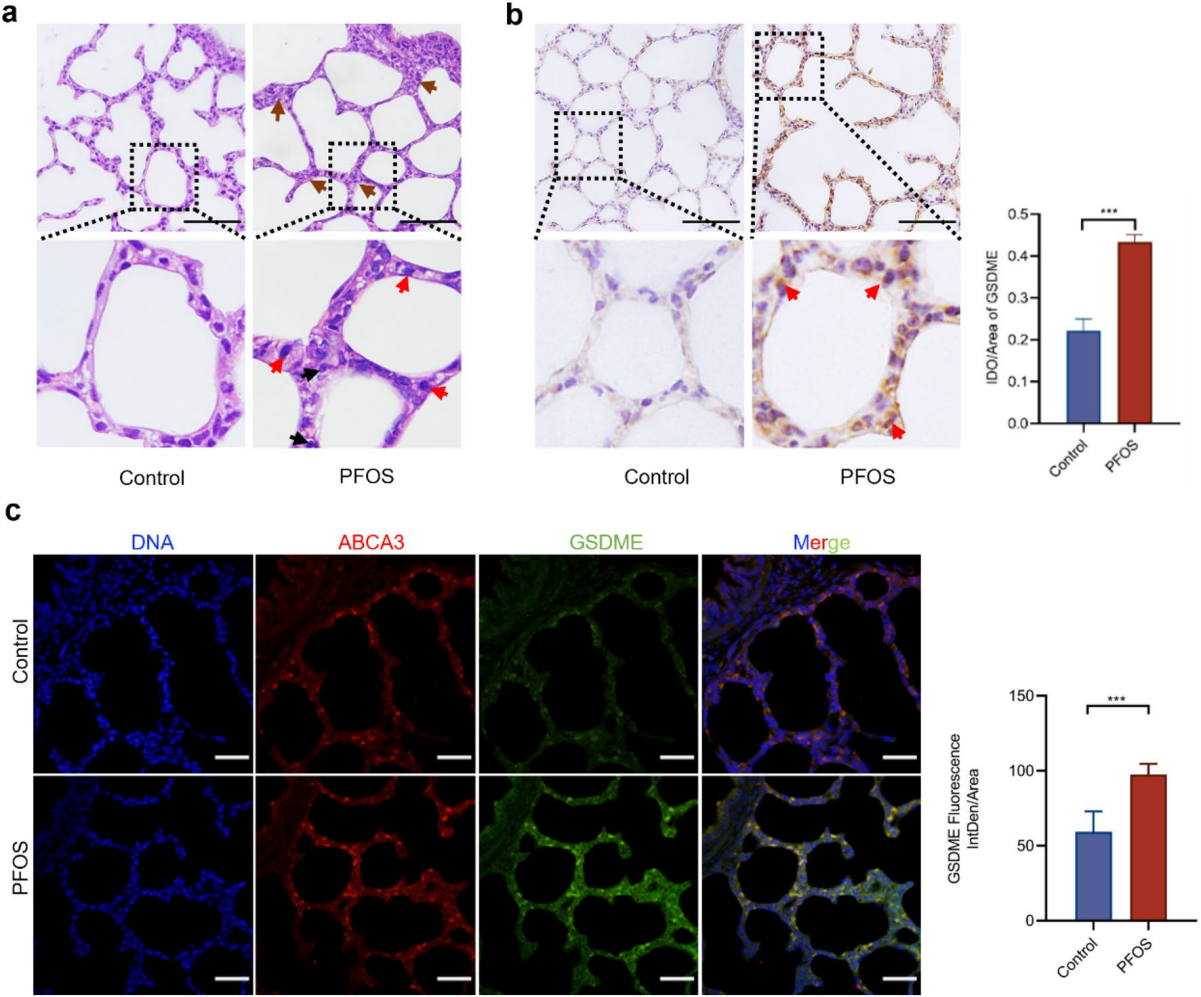
Gestational PFOS exposure induced lung inflammatory injury in offspring rats. SD rats were treated with either PFOS (1 mg/kg BW) or vehicle (0.5% Tween 20) from gestational day 12 to day 18. **a** Representative HE micrographs of PND7 lungs in offspring rats following PFOS exposure. Brown arrow: thickened alveolar septa; red arrow: macrophages; black arrow: neutrophils. Scale bar, 50 μm. **b** IHC analysis of GSDME. Red arrow: representative cells with GSDME positive staining, slides from 3 animals were analyzed (*n* = 3) for each group. **c** Immunofluorescence analysis of ABCA3 (red), GSDME (green), and DAPI (blue). For quantification of GSDME, slides from 3 animals were analyzed for each group. Scale bar, 20 μm. Data were presented as mean ± SD with statistical significances of ***p < 0.001

To investigate the impact of PFOS on fetal lung development, pregnant rats were exposed t PFOS (1 mg/kg BW) between gestational days 12 and 18. PFOS exposure led to an increase in the infiltration of inflammatory cells (Fig. 1a), thickened alveolar septa (Supplementary Fig. 1a), and elevated lung injury scores (Supplementary Fig. 1b). However, PFOS exposure did not affect the RAC score (relative alveolar counts) significantly (Supplementary Fig. 1c. Nevertheless, these results suggest that PFOS exposure during pregnancy affected offspring lung development.

Previous research has demonstrated a significant increase of a proinflammatory microenvironment in lung of PFOS exposed animals (Zhang et al. 2021). To further examine whether PFOS can induce inflammation-related cell death and pyroptosis, we examined the expression of GSDME and mRNA level of inflammatory factors *IL8*, *TNF-a* and *HMGB1* in PFOS exposed rats. As depicted in Fig. 1b, the number of GSDME-positive cells increased significantly in the lung of PFOS treated animal. The increased expression of GSDME-N and cleaved-caspase3 (Supplementary Fig. 1d) and mRNA level of *IL8*, *TNF-a* and *HMGB1* (Supplementary Fig. 1e) were also detected in the lung tissue of PFOS treated rats, suggesting possible activation of pyroptosis. Additionally, LDH levels in alveolar lavage fluid (Supplementary Fig. 1f) were elevated, providing further evidence that PFOS exposure during pregnancy induced pyroptosis in the lung of offspring rats. To examine whether AECII were involved in PFOS-induced pyroptosis, co-localization of GSDME and ATP-binding cassette transporter 3 (ABCA3), a marker for AECII (Sun et al. 2021) was analyzed (Fig. 1c). A significantly higher fluorescence intensity of GSDME was detected in AECII of PFOS animals than the controls, suggesting a potential AECII pyroptosis induced by PFOS.

### Effects of PFOS on AECII pyroptosis in vitro

**Fig. 2:**
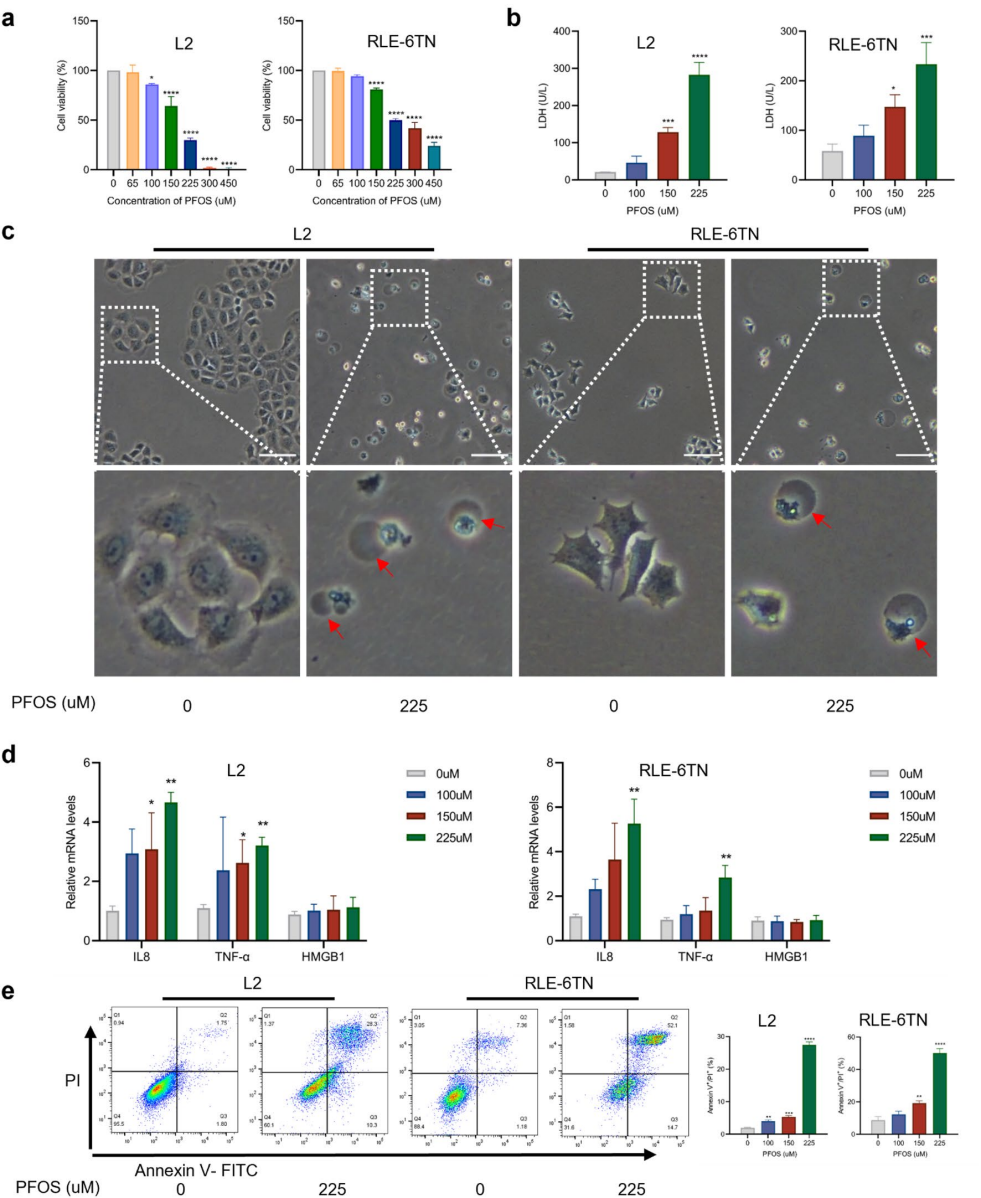
PFOS reduces cell viability by inducing pyroptosis in AECII. **a** Cells were exposed to various concentrations of PFOS (0, 65, 100, 150, 225, 300 and 450 μM) for 24 h and the viability was assessed using the CCK8 assay. **b** LDH levels were assayed for L2 and RLE-6TN after PFOS exposure. **c** Representative images of L2 and RLE-6TN cells after their treatment with different concentrations of PFOS for 24 h. Pyroptotic cells with bubble membrane were showed by red arrows. Scale bar, 100 μm. **d** The mRNA levels of pro-inflammatory cytokines *IL8*, *TNF-α* and *HMGB1* after PFOS exposure. **e** Percentage of pyroptosis cells in L2 and RLE-6TN cells (stained with Annexin V+/PI+). The data were presented as mean ± SD of three replicates (*n* = 3) with statistical significances of *p < 0.05, **p < 0.01,***p < 0.001 or ****p < 0.0001, respectively

To investigate the direct effects of PFOS on AECII, we conducted in vitro studies with RLE-6TN and L2 cell lines. Following exposure to various concentrations of PFOS for 24 h, there was a dose-dependent decrease in cell viability of both cell lines, based on CCK8 assay results (Fig. 2a). Further, the increase of LDH in the culture medium indicates a leakage of cellular content and a loss of membrane integrity in PFOS treated cells (Fig. 2b). Notably, at a concentration of 225 μM, the cells exhibited significant morphological alterations that deviated from the typical characteristics associated with apoptosis. These changes included cell swelling and the presence of large bubbles on the cell membrane (Fig. 2c and Supplementary Fig. 2a), which are distinct features of pyroptosis (Wang et al. 2017). In addition, PFOS treatment increased the mRNA levels of cellular inflammatory factors *IL8* and *TNF-α*, but not *HMGB1* in AECII (Fig. 2d). To further validate the presence of pyroptosis, we utilized flow cytometry using Annexin V-FITC and PI staining, which demonstrated a dose-dependent increase in the population of Annexin V+/PI+ cells in response to PFOS exposure (Fig. 2e and Supplementary Fig. 2b), unequivocally confirming the occurrence of pyroptosis.

### Involvement of GSDME in PFOS-induced AECII pyroptosis

**Fig. 3:**
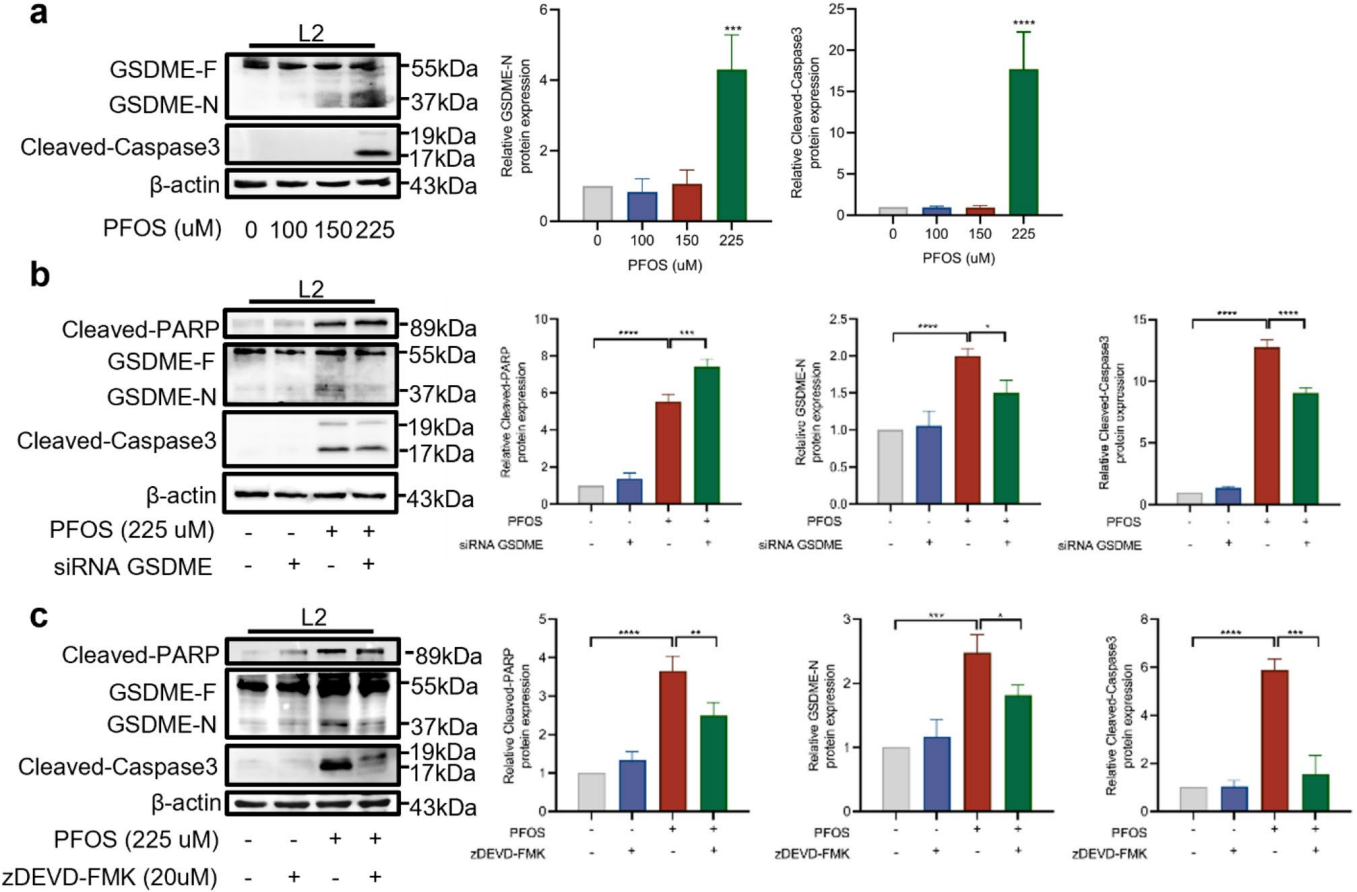
Involvement of GSDME and caspase-3 in PFOS induced AECII pyroptosis. **a** Cells were exposed to various concentrations of PFOS (0, 100, 150 and 225 μM) for 24 h. Proteins levels of GSDME-N and cleaved-caspase-3 were assessed using Western blot analysis. **b** Cells were transfected with siRNA-GSDME or siRNA-negative control and treated by PFOS (225 μM) for 24 h. Proteins levels of cleaved-PARP, GSDME-N and cleaved-caspase-3 were analyzed using Western blotting. **c** Western blot analyses of cleaved-PARP, GSDME-N and cleaved-caspase-3 after the cells were treated with caspase-3 inhibitor (zDEVD-FMK) and/or PFOS for 24h. The data were presented as mean ± SD of three replicates (n = 3) with statistical significances of *p < 0.05, **p < 0.01, ***p < 0.001, or ****p < 0.0001, respectively

Swelling and rupture of the cell membrane are key characteristics of GSDME-dependent pyroptosis. To explore more details of GSDME dependent pyroptosis in PFOS exposure, we assessed the levels of GSDME-N (a cleaved form of GSDME-F) following PFOS treatment. As shown in Fig. 3a and Supplementary Fig. 3a, treatment of both RLE-6TN and L2 cells with PFOS resulted in the accumulation of GSDME-F and appearance of GSDME-N. To further confirm the involvement of GSDME in PFOS-induced pyroptosis, we knocked down GSDME expression using siRNA. Transfection with GSDME siRNA reduced the expression levels of GSDME to 40.55% in L2 cells and 20.20% in RLE-6TN cells (Supplementary Fig. 3b). The number of cells with bubbles also significantly decreased in PFOS-treated GSDME knockdown cells compared to control cells, while non-lytic cell death was observed (Supplementary Fig. 3c). Additionally, the viability of cells with GSDME knockdown increased significantly in response to PFOS treatment, compared to the control groups (Supplementary Fig. 3d). Also, Western blotting analysis confirmed the effectiveness of GSDME knockdown (Fig. 3b and Supplementary Fig. 3e). Notably, GSDME knockdown resulted in reduced caspase-3 activation, generation of GSDME-N and the accumulation of cleaved PARP compared to cells transfected with negative siRNA controls (Fig. 3b and Supplementary Fig. 3e), suggesting the potential involvement of GSDME in PFOS-induced pyroptosis.

### Caspase-3 is indispensable for PFOS induced AECII pyroptosis

Pyroptosis requires the involvement of caspase, with caspase-3 being essential for GSDME dependent pyroptosis. To explore the potential role of caspase-3 in PFOS-induced pyroptosis, we assessed the levels of activated (cleaved) caspase-3 protein following PFOS treatment. As shown in Fig. 3a and Supplementary Fig. 3a, treatment of both RLE-6TN and L2 cells with PFOS resulted in the generation of the active forms of caspase-3. Additionally, treatment with caspase-3 inhibitor zDEVD-MK resulted in a significant increase in cell viability (Supplementary Fig. 4a), along with noticeable changes in cell morphology (Supplementary Fig. 4b). Inhibition of caspase-3 also led to a reduction in the production of activated caspase- and effectively hindered most of the GSDME cleavage (Fig. 3c and Supplementary Fig. 4c), indicating that PFOS may induce AECII pyroptosis through the activation of caspase-3/GSDME pathway.

### PFOS activates ER stress and PERK/ATF4 signaling in AECII

**Fig. 4:**
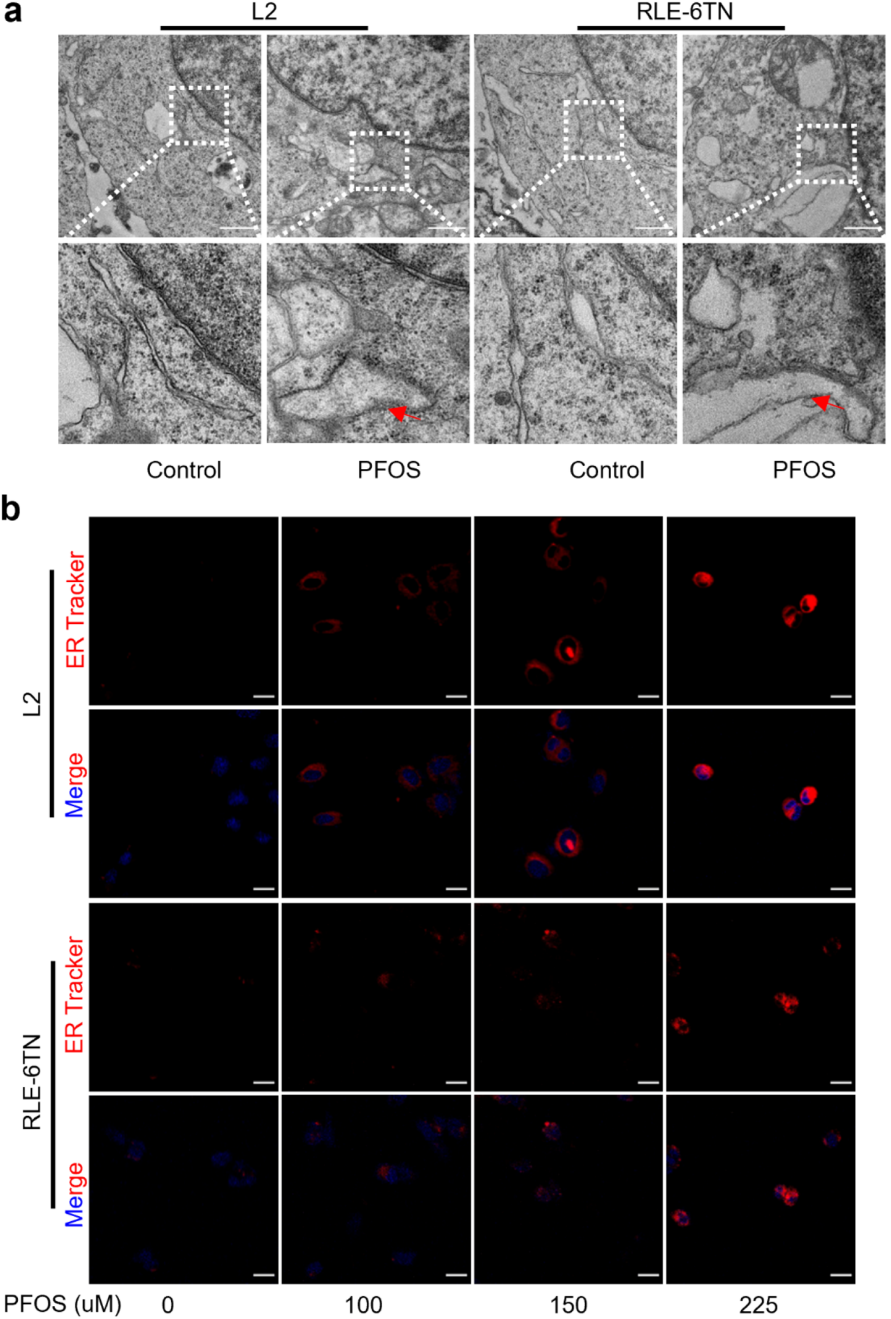
Effect of PFOS on ER stress in AECII. **a** The ultrastructure changes of ER of cells following treatment with various concentrations of PFOS (0 and 225 μM) for 24 h. Inflated and swollen ER are indicated by red arrows. Scale bar, 1 μm. **b** Swollen dysfunctional ER levels were evaluated by ER Tracker (red). Scale bar, 20 μm

Studies have confirmed that PFOS-induced cell death can be triggered by the ER stress (Zhang et al. 2023). To investigate whether ER stress is associated with PFOS-induced GSDME-dependent pyroptosis, we used TEM to examine the ultrastructural changes in the ER of AECII undergoing pyroptosis. As depicted in Fig. 4a, PFOS-treated cells displayed disordered and swollen ER structures with a vesicular appearance, contrasting with the morphology of cells in the negative control group. Moreover, the fluorescence intensity of ER tracker increased in a concentration-dependent manner upon PFOS exposure (Fig. 4b and Supplementary Fig. 5a), providing further confirmation that PFOS affected ER structure and function in AECII.

**Fig. 5:**
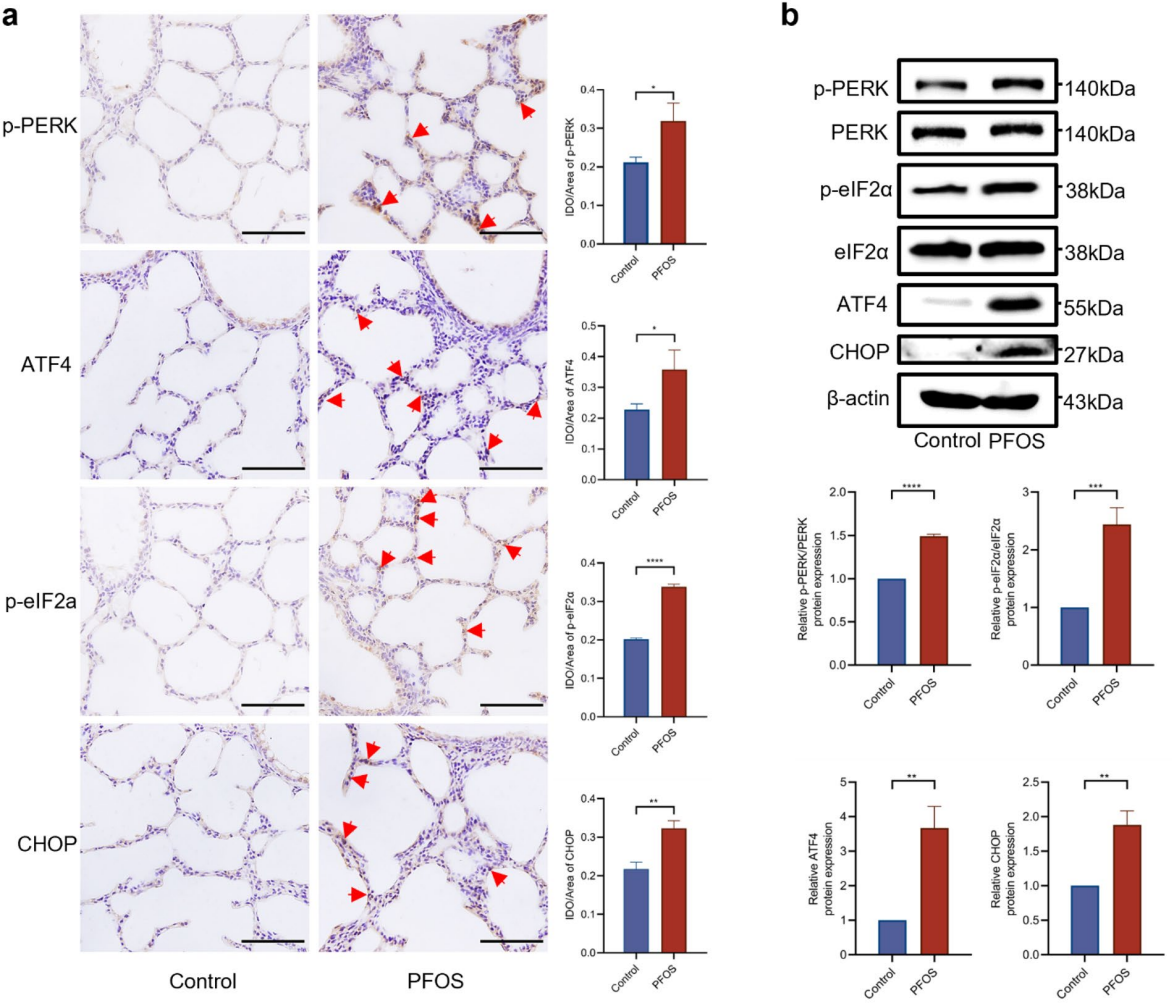
Effect of PFOS on ER stress-mediated PERK/ATF4 signaling in lung tissues. **a** IHC analysis of p-PERK, p-eIF2α, ATF4 and CHOP in rat lungs with or without PFOS treatment. Red arrows: positive cells. Scale bar, 50 μm. Slides from 3 animals were analyzed (*n* = 3) for each group. **b** The protein levels of p-PERK, p-eIF2α, ATF4 or CHOP of rat lungs were assessed by western blotting. The data were presented as mean ± SD with statistical significances of *p < 0.05, **p < 0.01, ***p < 0.001, or ****p < 0.0001, respectively

**Fig. 6:**
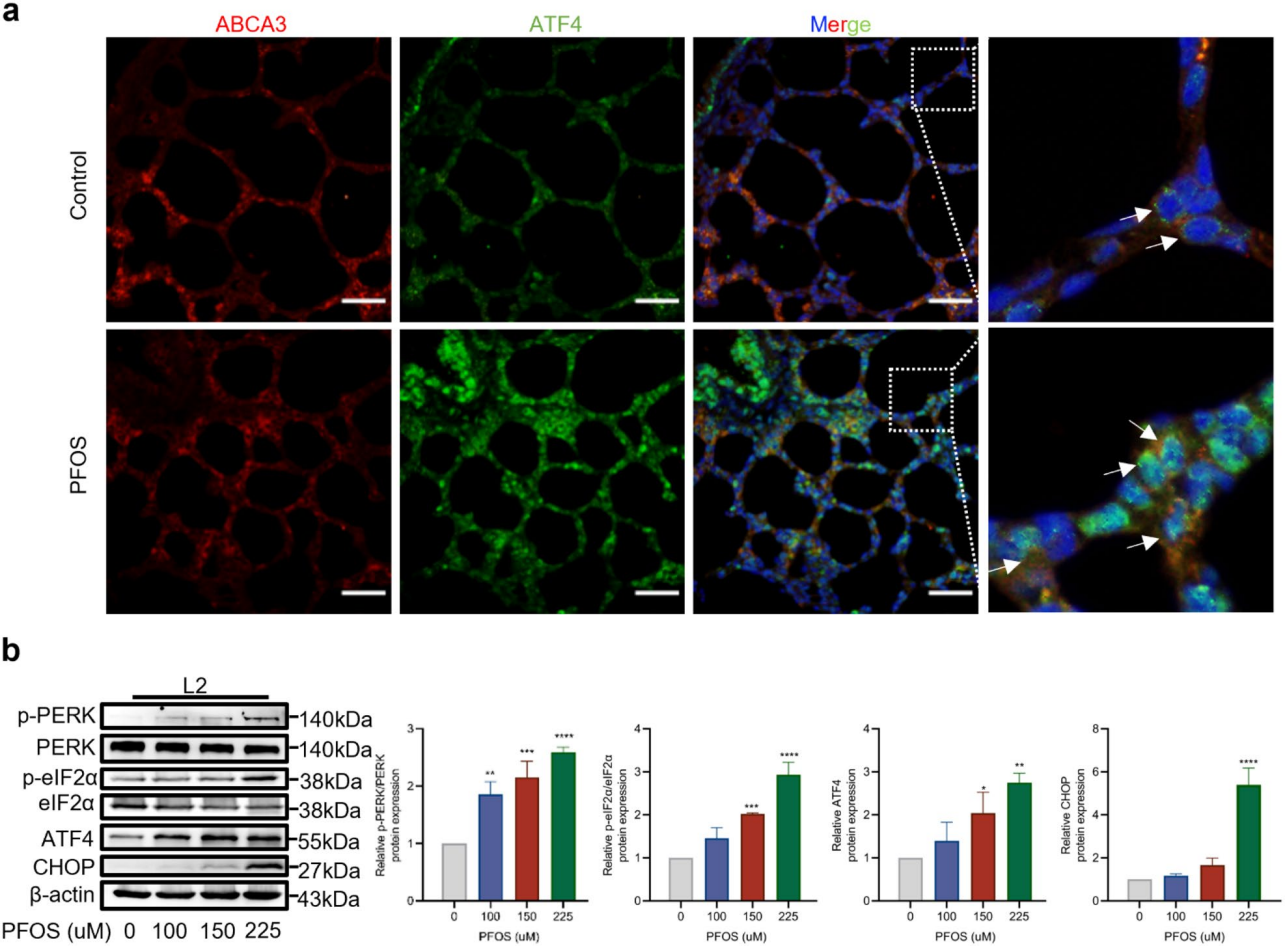
Effect of PFOS on ER stress-mediated PERK/ATF4 signaling in AECII. **a** Effect of PFOS on the expression of ABCA3 (red), ATF4 (green), and DAPI (blue) in vivo (PND7). White arrow: AECII. Scale bar, 20 μm. **b** The protein levels of p-PERK, p-eIF2α, ATF4 or CHOP were assessed by western blotting. The data were presented as mean ± SD of three replicates (*n* = 3) with statistical significances of *p < 0.05, **p < 0.01, ***p < 0.001, or ****p < 0.0001, respectively

To examine the involvement of key ER stress signaling molecules, including *ATF4*, *ATF6*, and *XBP1*, we conducted qPCR to assess their mRNA levels. PFOS exposure led to a dose-dependent increase in *ATF4* expression, while *ATF6* and *XBP1* were only affected at highest PFOS concentration (Supplementary Fig. 5b). Furthermore, IHC analysis and western blotting analysis of lung tissue demonstrated significantly higher expression of p-PERK, p-eIF2α, ATF4, and CCAAT/enhancer-binding protein homologous protein (CHOP) in PFOS group (Fig. 5a and b). Immunofluorescence colocalization analysis further confirmed the elevated expression of ATF4 specifically in AECII (Fig. 6a). Additionally, Western blotting analysis demonstrated a dose-dependent upregulation of ER stress markers (p-PERK, p-eIF2α, ATF4, and CHOP) in AECII upon PFOS treatment (Fig. 6b and Supplementary Fig. 5c), suggesting their involvements in PFOS-induced pyroptosis. Interestingly, expressions of both cytoplasmic GSDME and nuclear ATF4 increased following PFOS treatment (Supplementary Fig. 5d), suggesting the activation of ATF4 by PFOS exposure.

### Activation of PERK signaling pathway triggers GSDME mediated pyroptosis in response to PFOS

**Fig. 7:**
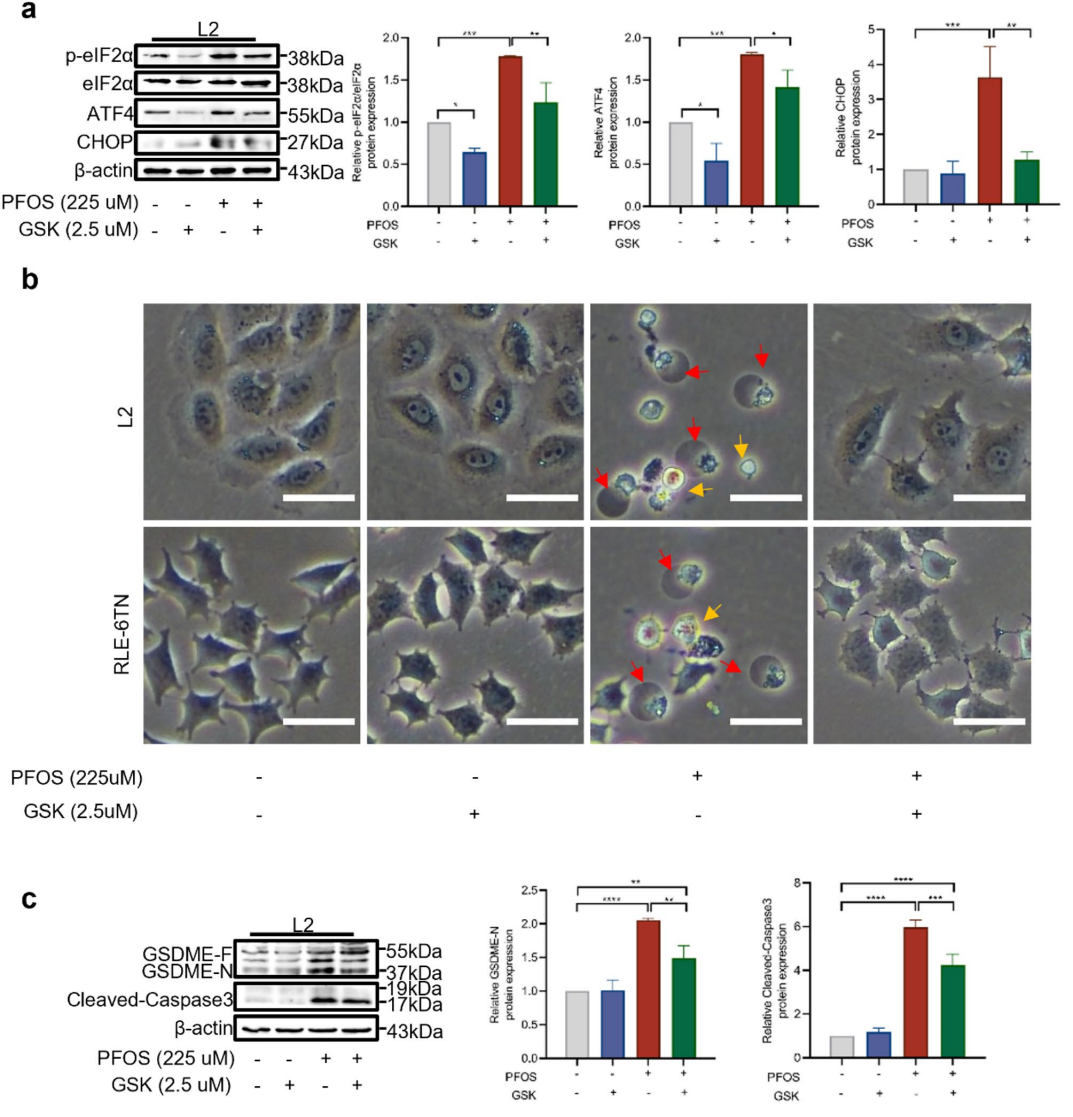
Involvement of PERK/ATF4 signaling in GSDME-dependent AECII pyroptosis induced by PFOS. Cells were incubated with GSK (2.5 μM) and/or PFOS (225 μM) for 24 h. **a** Western blot analyses of p-eIF2α, ATF4 and CHOP. **b** Representative pyroptotic (red arrow) and apoptotic (yellow arrow) morphology of AECII treated with GSK and/or PFOS. Scale bar, 50 μm. **c** Western blot analyses of cleaved-PARP, GSDME-N or cleaved-caspase-3. Data were presented as mean ± SD of three replicates (*n* = 3) with statistical significances of *p < 0.05, **p < 0.01, ***p < 0.001, or ****p < 0.0001, respectively

To elucidate the role of PERK in PFOS-induced GSDME-dependent pyroptosis, we utilized GSK2606414 (GSK), a specific inhibitor of PERK phosphorylation, to investigate its impact. Treatment with GSK (2.5 μM) resulted in a reduction in ER tracker fluorescence intensity in PFOS-exposed AECII (Supplementary Fig. 6a). Moreover, GSK treatment resulted in reduced protein levels of ATF4 and CHOP, along with a decrease in the p-eIF2α/eIF2α ratio (Fig. 7a and Supplementary Fig. 6b). Importantly, GSK treatment also reduced intracellular fluorescence intensity of GSDME and ATF4 in AECII (Supplementary Fig. 6c), indicating the effective inhibition of PERK-related ER stress induced by PFOS. Furthermore, co-treatment with GSK and PFOS resulted in increased cell viability (Supplementary Fig. 6d), alterations in cell morphology (Fig. 7b), a reduction in the proportion of Annexin V+/PI+ cells (Supplementary Fig. 7a), and reduced LDH release (Supplementary Fig. 7b), suggesting that PERK-associated ER stress plays a crucial upstream role in PFOS-induced pyroptosis. Simultaneously, GSK treatment decreased the high mRNA levels of cellular inflammatory factors IL8 and TNF-α induced by PFOS in AECII (Supplementary Fig. 7c). Interestingly, both PFOS and GSK failed to affect HMGB1 levels significantly. Western blot analysis confirmed that GSK partially inhibited the PFOS- induced formation of cleaved caspase-3 and GSDME-N (Fig. 7c and Supplementary Fig. 7d), which is consistent with the results obtained from flow cytometry and immunofluorescence results.

## Discussion

PFOS pollution has emerged as a potential threat to human health (Ahmed et al. 2020). Concerns about its role in early human development are mounting, since PFOS has been consistently detected in the blood of pregnant women (Callan et al. 2016), and in more than 99.7% of umbilical cord blood samples (Cao et al. 2018; Tsai et al. 2017). Neonatal bronchopulmonary dysplasia is one of developmental disorders associated with high mortality rates in newborns. It is characterized by severe respiratory compromise due to a reduction in the number of pulmonary alveoli or bronchial branching (Gilfillan et al. 2021). The exact cause of this disorder is not fully understood, but environmental factors, such as PFOS, may potentially contribute to its development (Liang et al. 2022).

Human lung development comprises five periods: embryonic, pseudoglandular, canalicular, saccular and alveolar stages. The pseudoglandular stage in humans is within weeks 7–17 of gestation, which corresponds to gestational days 12–18 in rats. During the pseudoglandular stage, the bronchial and vascular systems of the fetal lung undergo further branching, and epithelial cells begin to differentiate and form cartilages, smooth muscles, and the first gland-like structures (Mullassery and Smith 2015). The result of our study indicates that exposure to PFOS during this critical period resulted in alveolar septal thickening and inflammatory infiltration in the lungs, which is consistent with previous findings (Chen et al. 2012; Grasty et al. 2003; Mao et al. 2013). The elevated mRNA levels of early pro-inflammatory cytokines *IL8* and *TNF-α*, along with late-stage pro-inflammatory cytokine *HMGB1*, suggest that prenatal exposure to PFOS led to lung inflammation, which may potentially persist. However, in vitro experiments showed no significant difference in HMGB1, which may be attributed to the relatively short 24-h exposure period or AECII not being a major producer of the factor.

Interestingly, Dragon et al. (2023) did a similar in vitro experiment with a bronchial epithelial cell line (BEAS2B), and found a significant increase in HMGB1 following PFOS treatment. The difference between the two studies is still clear, but could be due to variations in cell types and/or species. Another unexpected observation is the failure to detect a significant reduction in RAC score. The reason could be that the lungs are still in the early stages of alveolarization at PDN7, with the transformation of alveolar walls into mature structures yet to be completed, and the final alveolar architecture yet to be fully developed (Qing et al. 2022). Future studies are needed to check lung maturation and RAC scores in the later ages after PFOS exposure.

One of the key features for pseudoglandular stage is a differentiation of the first AECII at the distal part of the lung (Lewin and Hurtt 2017). AECII plays a critical role in lung development and function by serving as epithelial progenitor cells in maintaining the integrity of alveolar epithelium and facilitating tissue regeneration (Hogan et al. 2014). In the event of lung injury, these cells possess the ability to differentiate into alveolar type I epithelial cells, which is a crucial mechanism for tissue repair (Evans and Lee 2020). Furthermore, AECIIs are responsible for surfactant secretion, a process crucial for reducing surface tension at the alveolar gas–liquid interface and maintaining optimal lung function (Wright and Hawgood 1989). Therefore, ensuring the integrity and function of AECII is of paramount importance for repairing alveolar structures and maintaining overall lung health. In this study, we observed that PFOS exposure during pregnancy resulted in increased expression of GSDME in AECII of the offspring rats. Furthermore, consistent with these findings, our in vitro experiments demonstrated that PFOS has the potential to induce pyroptosis in AECII, suggesting a potential mechanism by which PFOS influences lung development in vivo.

Various forms of cell death have been identified in recent years. However, that signaling pathways governing these different processes are not entirely independent, instead, they often intersect and exert influence on one another (Tang et al. 2019). Caspase-3 plays a central role in cleaving numerous cellular substrates and is a major intersection point for multiple forms of cell death (Eskandari and Eaves 2022), including the GSDME-dependent pyroptosis (Wang et al. 2017). Previous studies have shown that PFOS can activate caspase-3 and subsequently, triggers the cleavage of PARP, thus inducing cellular apoptosis (Han et al. 2018; Tang et al. 2022). In this study, we expanded this conventional perspective and discovered that caspase-3 activation due to PFOS exposure can also trigger pyroptosis, as confirmed by zDEVD-FMK experiments. Prior research has established that the level of GSDME plays a crucial role in determining which form of cell death could be induced (Wang et al. 2017). Cells with elevated GSDME expression undergo pyroptosis upon caspase-3 activation, while cells with low GSDME expression undergo apoptosis (Liu et al. 2022; Wang et al. 2022a; Yu et al. 2019). In line with these findings, our study demonstrated that PFOS primarily induced pyroptosis by up-regulation of GSDME. Knocking down of GSDME only partially prevented cell death and the disappearance of the cell membrane bubble, a typical character of pyroptosis cells. Interestingly, accompanied the reduction in pryoptosis, cells with apoptotic morphology and PARP expression increase, suggesting a potential transition from pyroptosis to apoptosis.

Multiple lines of evidence have indicated that ER stress may participate in the PFOS-related cellular injuries and tissue damages, especially for those related to PERK/ATF4 signaling pathway. PFOS was found to upregulate the expression of 78-kDa glucose-regulated protein (GRP78) in the cerebral cortex, leading to neuronal apoptosis (Oh et al. 2018). Meanwhile, PFOS could selectively activate the PERK/CHOP signaling pathway, in the cerebral cortex of mice, thereby inducing cognitive dysfunction (Zhang et al. 2023). Furthermore, the PERK/ATF4 related ER stress may be associated with high cellular triglyceride levels in damaged liver cells induced by PFOS (Louisse et al. 2020). Therefore, we investigated whether ER stress is involved in PFOS-induced lung injury in the current study. A hallmark of ER stress is the disordered structure and vesicular dilation of the ER (Wang et al. 2021). Our results of TEM and ER-Tracker provide evidence that PFOS exposure indeed leads to ER swelling. The ER stress response involves three primary signaling sensors, including PERK, inositol-requiring protein 1α (IRE1α), and ATF6 (Chen et al. 2018). Among these, PERK serves as an immediate early response sensor (Wang et al. 2021). Activated PERK then phosphorylates eIF2α, leading to a general inhibition of translation in the early stages of ER stress. Subsequently, downstream ATF4 promotes the expression of the apoptosis-inducing factor CHOP in response to prolonged ER stress (Celik et al. 2023). In this study, we found the involvement of PERK/ATF4 signaling pathway. The levels of p-PERK, p-eIF2α, ATF4 and CHOP were all significantly up-regulated in AECII after PFOS exposure. Immunohistochemical data also support the upregulation of ER stress proteins upon PFOS exposure.

The connection between ER stress and cell pyroptosis has been studied in other systems. For instance, methamphetamine induced GSDME-dependent cell death in hippocampal neuronal cells by ER stress signaling (Liu et al. 2020). Myricetin, another ER stress-inducing substance, can increase cleaved caspase-3 and GSDME-N by up-regulating ROS and caspase-12 levels, leading to pyroptosis of lung cancer cells (Han et al. 2022). In the current study, use of PERK phosphorylation inhibitor GSK significantly reduced the number of AECII with pyroptotic morphology, lowered the ratio of Annexin V+/P+ cells, and resulted in a higher proportion of Annexin V+ apoptotic cells. However, while GSK partially inhibited the PFOS-induced increase in the ratio of Annexin V+/PI+ cells and the cleavage of GSDME, it was unable to completely prevent these events. This suggests the involvement of additional signaling molecules, beyond PERK, in PFOS-induced pyroptosis. Considering the intricate interplay between PFOS-induced pyroptosis and apoptosis, further investigations are necessary to explore the roles of other signaling molecules involved in the interactions between ER stress, caspase-3/GSDME and apoptosis/pyroptosis cascades.

## Conclusions

**Fig. 8:**
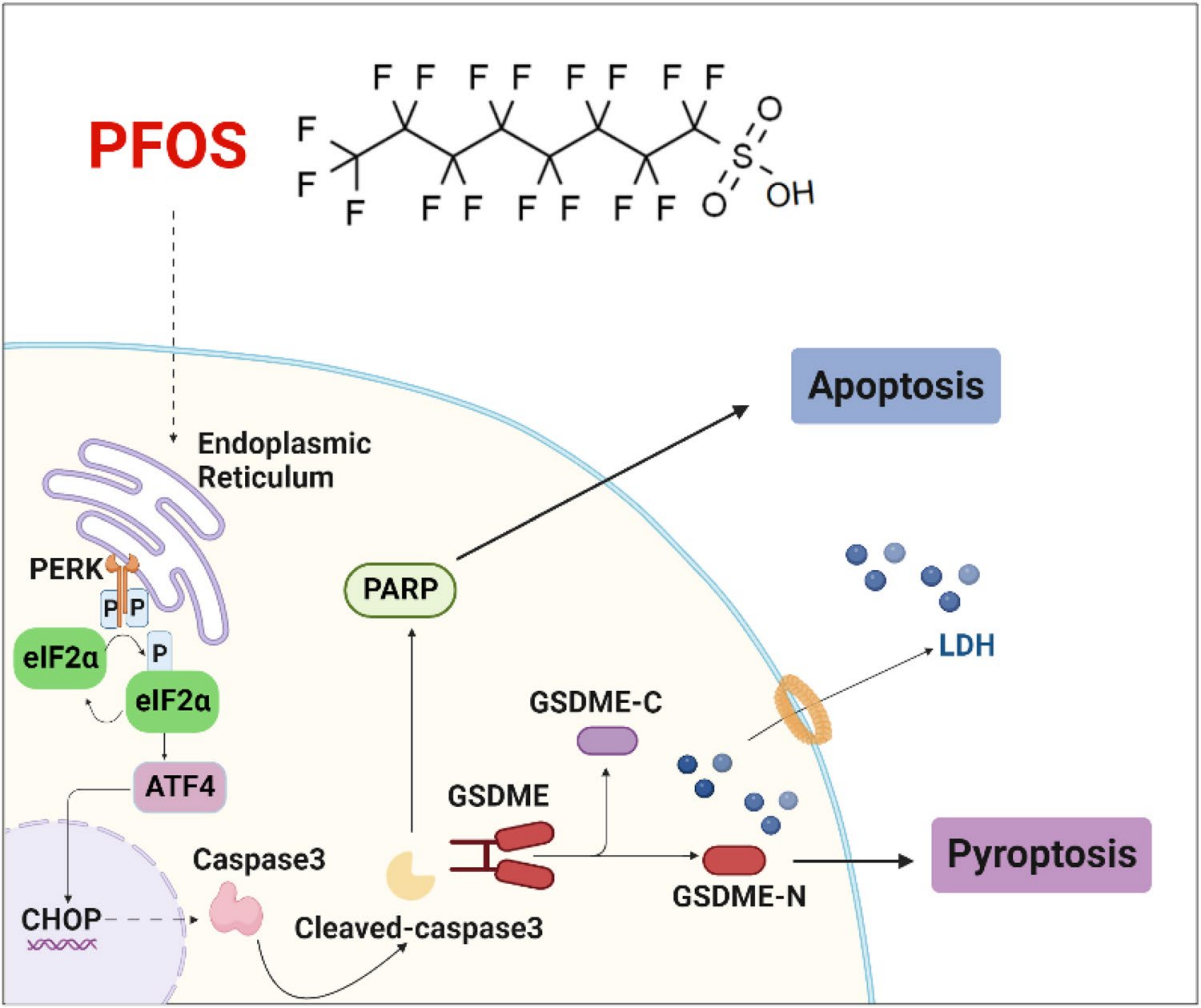
Schematic diagram to illustrate the mechanism by which PFOS-induces caspase-3/GSDME-dependent ACEII pyroptosis in offspring rats. PFOS induces lung damage by up-regulating PERK/ ATF4 signaling and activating caspase-3 to cleavage GSDME to produce GSDME-N, triggers ACEII pyroptosis

In conclusion, our study uncovers that gestational PFOS exposure can lead to lung injury in offspring, possibly by increasing AECII pyroptosis through activating the GSDME and ER stress-related PERK/ATF4 signaling pathway (Fig. 8). The study provides evidence for a potential link between gestational PFOS exposure and neonatal pulmonary hypoplasia. Also, it identifies ER stress and related signaling molecules PERK/ATF4 as potential mechanism involved, which may also function as a promising therapeutic target for preventing such effect.

### Supplementary Information

Below is the link to the electronic supplementary material.

Supplementary file1 (DOCX 8432 KB)

## Data Availability

All data supporting the findings of this study are available within the paper and its Supplementary Information. Raw data are available from the corresponding author upon requests.
