# Supplementary material for: Caspase-3/GSDME dependent pyroptosis contributes to offspring lung injury induced by gestational PFOS exposure via PERK/ATF4 signaling
Source: Arch Toxicol. 2023 Nov 13;98(1):207–21. doi: 10.1007/s00204-023-03626-w (PMC10761489; doi:10.1007/s00204-023-03626-w)
Supplement: Supplementary file 1 — Supplementary file1 (DOCX 8432 KB) [file 204_2023_3626_MOESM1_ESM.docx]

**Caspase-3/GSDME dependent pyroptosis contributes to offspring lung injury induced by gestational PFOS exposure via PERK/ATF4 signaling**

Cong Li^1,#^，Huishan Zhang^1,2,#^，Jiali Mo^1^，Jingye Zuo^1^，Leping Ye^1,*^

^1^ Department of Pediatrics, Peking University First Hospital, Beijing, 100034, China.

^2^ Department of Respiratory, Shanghai Children’s Medical Center, School of Medicine, Shanghai Jiao Tong University, Shanghai, 200120, China.

^*^Corresponding author: Leping Ye, Department of Pediatrics, Peking University First Hospital, No.1 Xi'an Men Street, West District, Beijing, 100034, China.

E-mail: yeleping@bjmu.edu.cn, [yeleping@163.com](mailto:yeleping@163.com).

**Supplementary Figures**


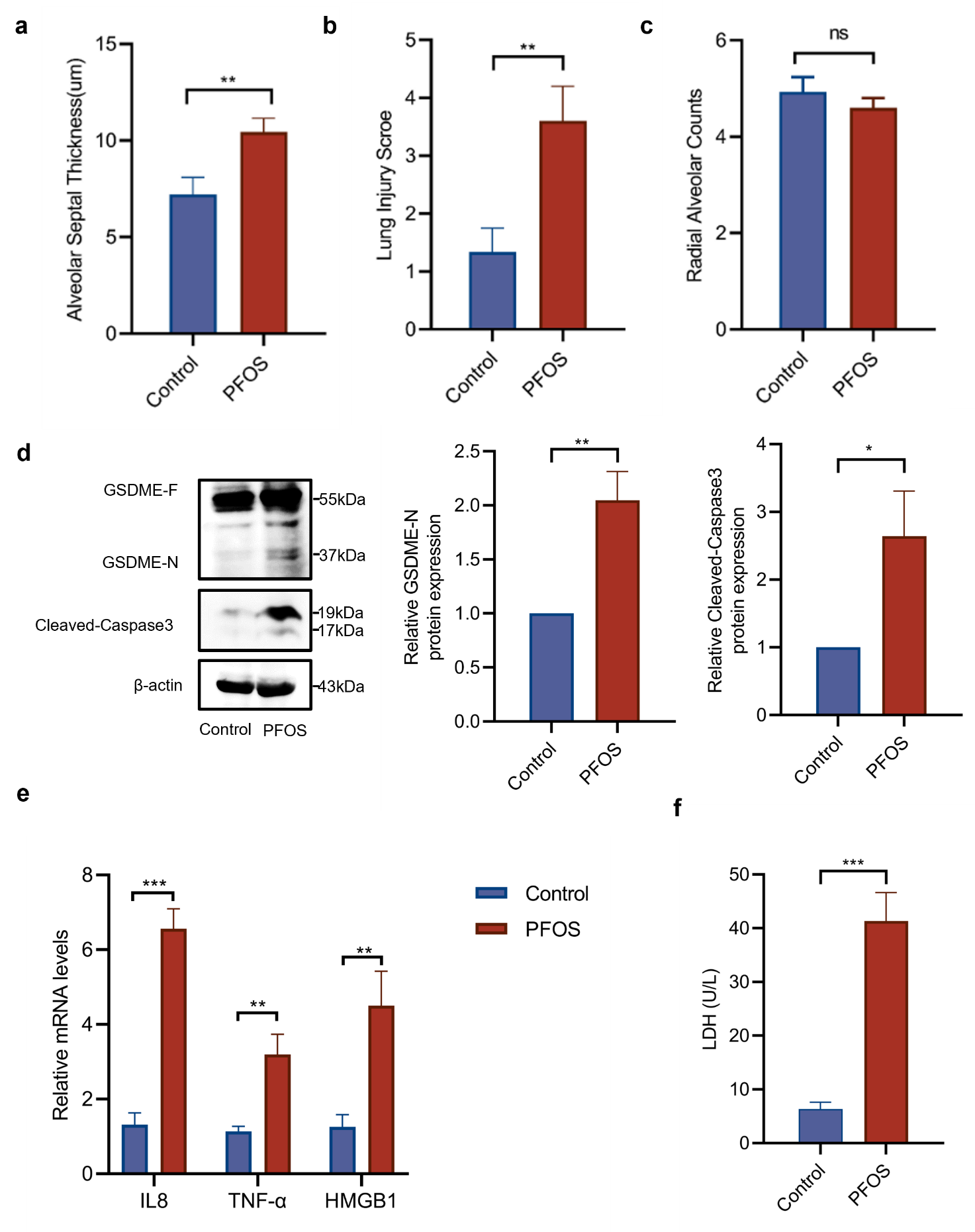


**Supplementary Fig. 1** Effects of PFOS on lung structure, injury score and pyroptosis. **a** alveolar septal thickness. **b** Smith lung injury score. **c** radial alveolar counts. Slides from 3 animals were analyzed (n=3) for each group. **d** The levels of GSDME-N and cleaved-caspase-3 proteins were analyzed determined by Western blots and expressed as ratio of controls. **e** RNA expression of 3 inflammatory factors. **f** LDH level of alveolar lavage fluid. Scale bar, 50 μm. Data is presented as mean±SD of three replicates (n=3) with statistical significances of * *p*<0.05, ** *p*<0.01, *** *p*<0.001.
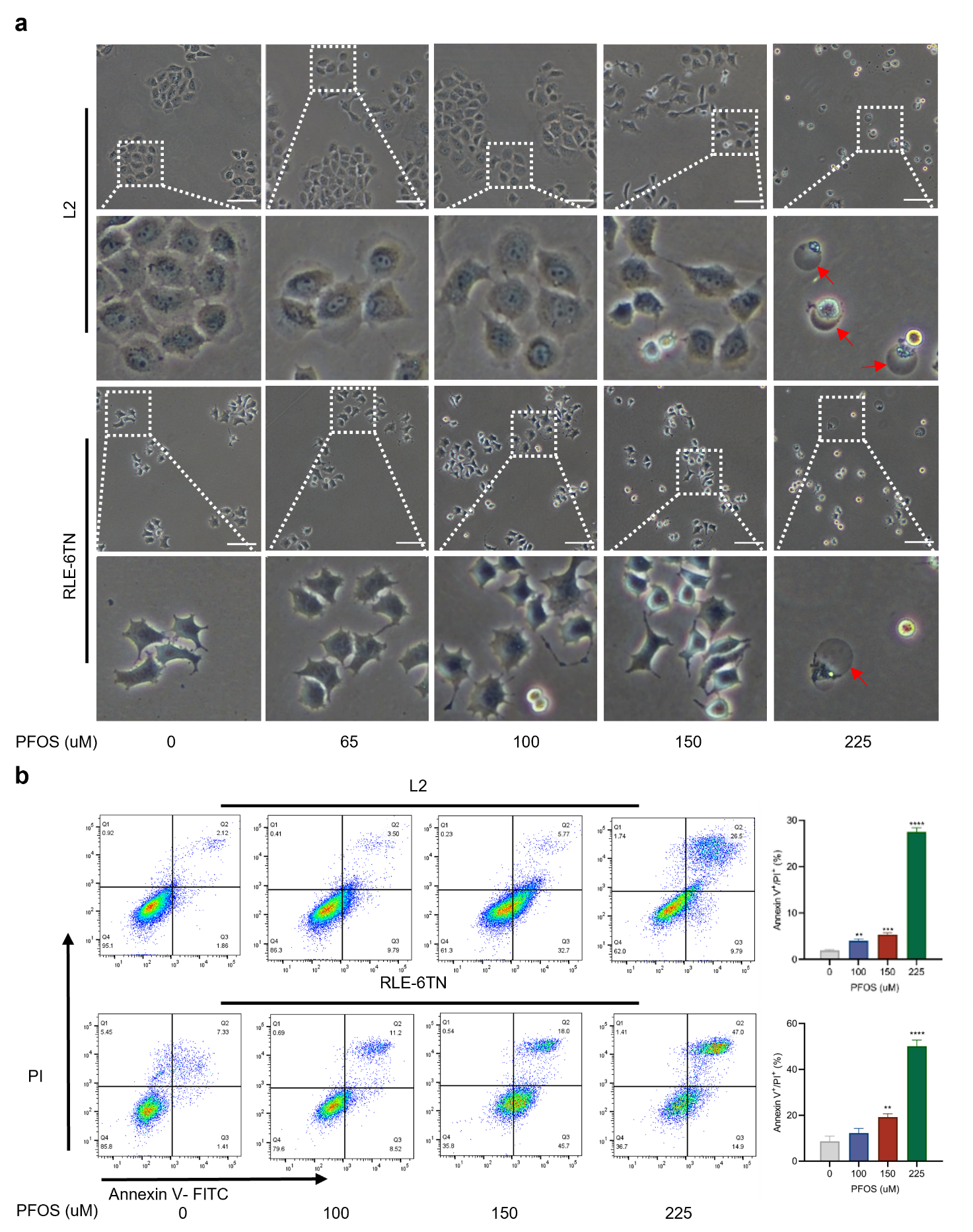


**Supplementary Fig. 2** PFOS inhibits cell viability by inducing pyroptosis in AECⅡ. **a** Representative bright-field microscopy images of cells that treated with different concentrations (0, 65, 100, 150, and 225 μM) of PFOS for 24 h. Pyroptotic cells with membrane bubble were showed by red arrows. Scale bar, 100 μm. **b** Percentage of L2 and RLE-6TN cells with pyroptotic characteristic (stained with Annexin V+/PI+). The data is presented as mean±SD of three replicates with statistical significances of * *p*<0.05, ** *p*<0.01, *** *p*<0.001 or *****p*<0.0001, respectively.


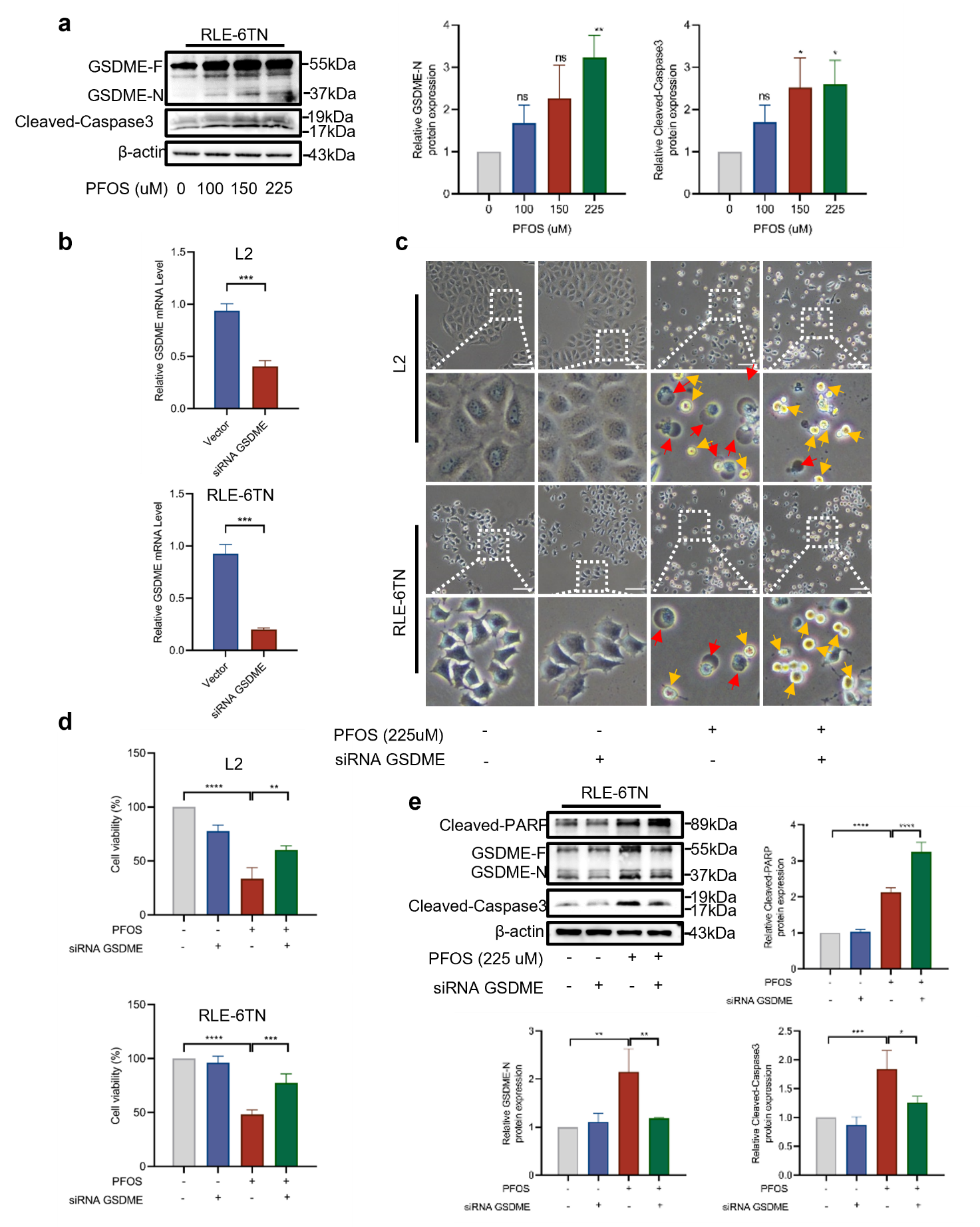


**Supplementary Fig. 3** Involvement of GSDME in PFOS induced AECII pyroptosis. **a** Western blot analyses of GSDME-N and cleaved-caspase-3 of cells treated with PFOS for 24h. **b** Cells were transfected with siRNA-GSDME or siRNA-negative control and treated by PFOS (225μM) for 24 h. The mRNA level of GSDME in cells was determined by qPCR. **c** Representative microscopy images of cells under the intervention of zDEVD-FMK (20μM). red arrow: pyroptotic cell; yellow arrow: apoptotic cells. Scale bar, 100 μm. **d** Cell viability was measured after treated with or without zDEVD-FMK (20μM) and PFOS (225μM) for 24 h. **e** Proteins levels of cleaved-PARP, GSDME-N and cleaved-caspase-3 were determined by Western blots. The data were presented as mean±SD of three replicates (n=3) with statistical significances of * *p*<0.05, ** *p*<0.01, *** *p*<0.001, or **** *p*<0.0001, respectively.


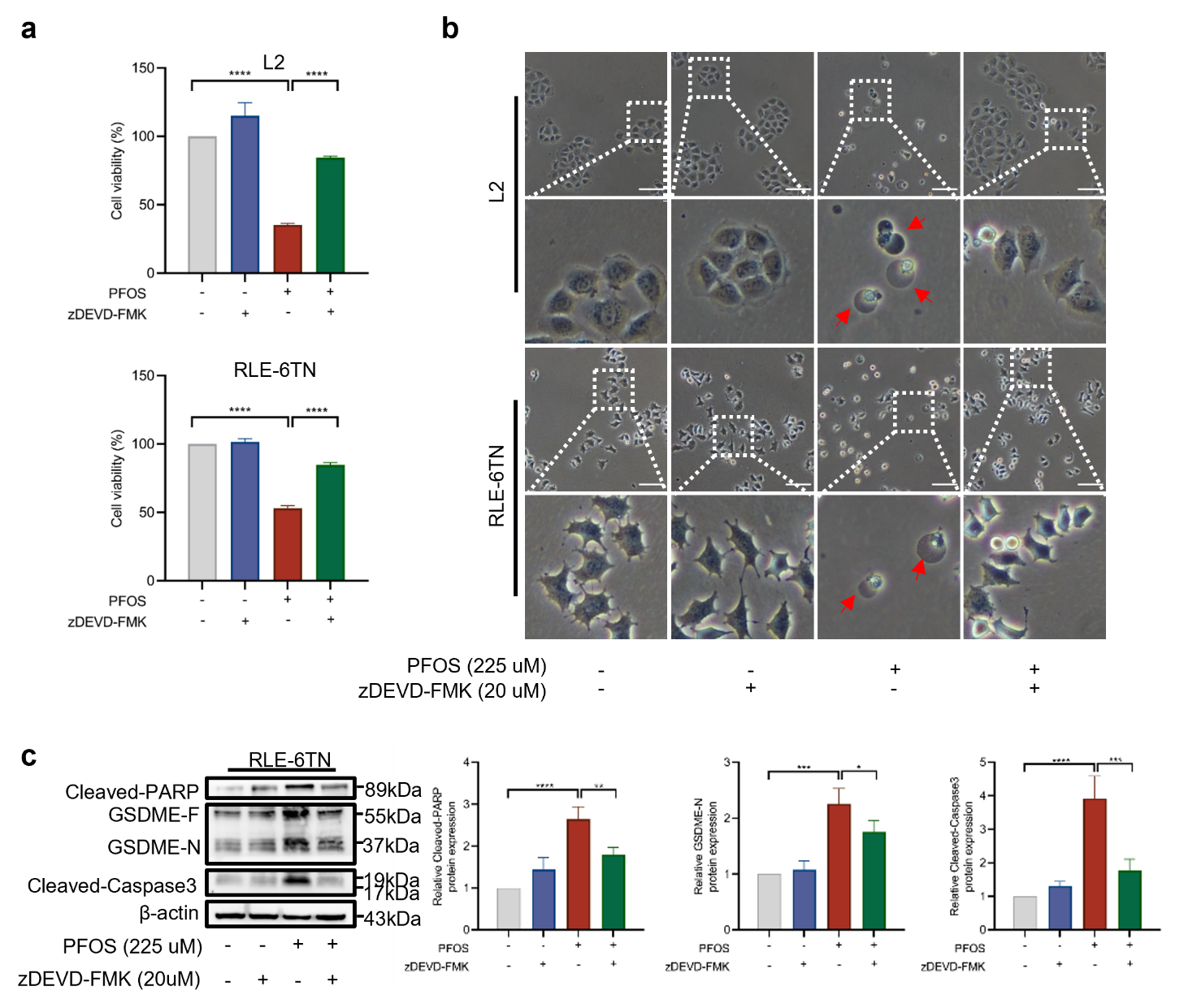


**Supplementary Fig. 4** Caspase-3 mediated PFOS-induced AECⅡ pyroptosis. **a** Cell viability was measured after treated with or without caspase 3 inhibitor zDEVD-FMK (20μM) and PFOS (225μM) for 24 h. **b** Representative microscopy images of cells, red arrows: pyroptotic cells. Scale bar, 100 μm. **c** Western blot analyses of cleaved-PARP, GSDME-N and cleaved-caspase-3 treated with zDEVD-FMK and/or PFOS for 24h. The data were presented as mean±SD of three replicates (n=3) with statistical significances of * *p*<0.05, ** *p*<0.01, *** *p*<0.001, or **** *p*<0.0001, respectively.


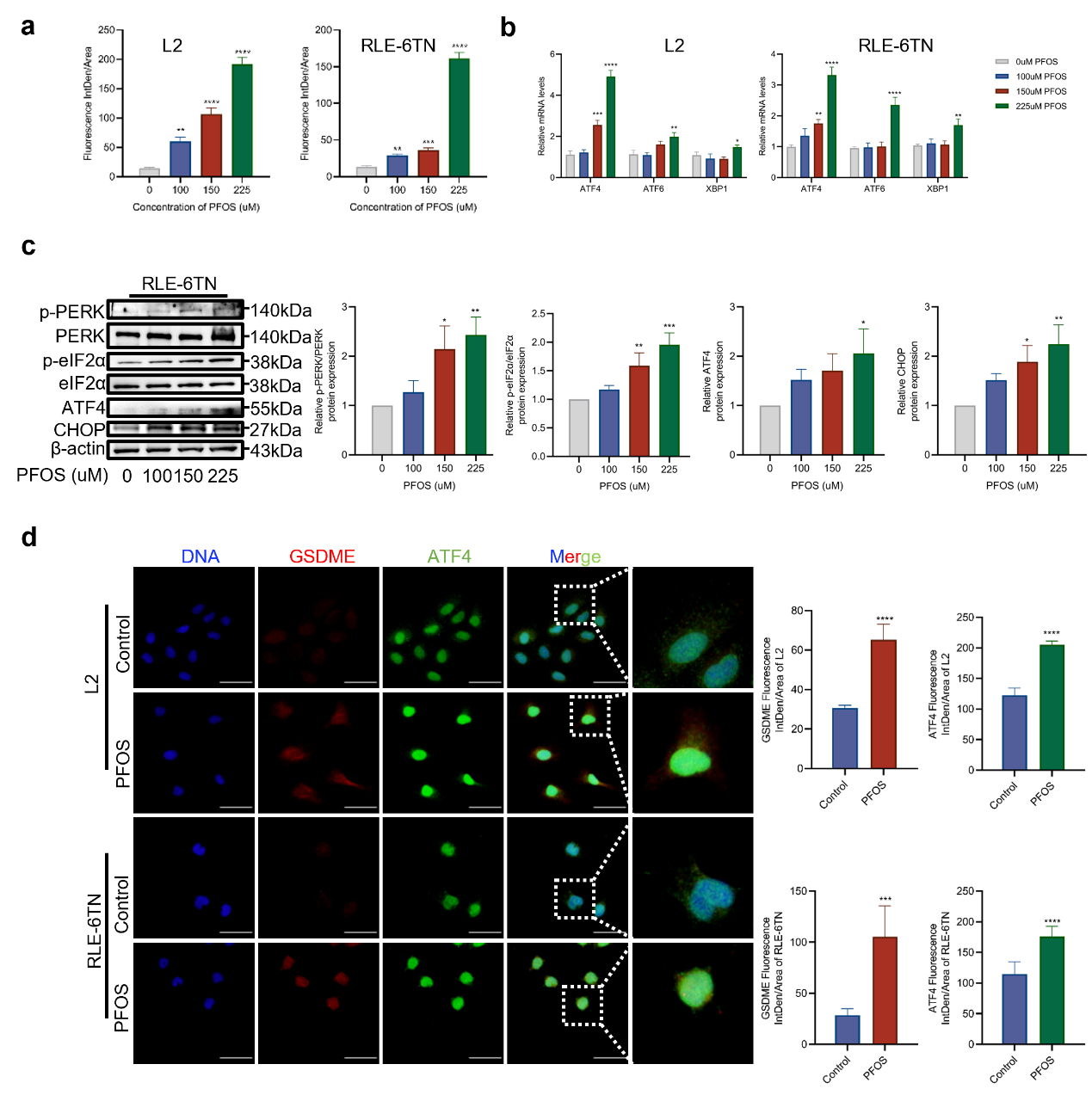


**Supplementary Fig. 5** Involvement of ER stress in PFOS-induced PERK/ATF4 signaling in AECⅡ. **a** The fluorescence intensity of swollen ER levels. **b** mRNA levels of ER stress-related genes in L2 and RLE-6TN cells with exposure of PFOS (0, 100 ,150 and 225μM). **c** The protein levels of p-PERK, p-eIF2α, ATF4 or CHOP were assessed by Western blotting. **d** Expression of GSDME (red), ATF4 (green) and DAPI (blue) after the cells were exposed with PFOS (0 and 225μM）for 24h. Scale bars, 20 μm. The data were presented as mean±SD of three replicates (n=3) with statistical significances of * *p*<0.05, ** *p*<0.01, *** *p*<0.001, or **** *p*<0.0001, respectively.


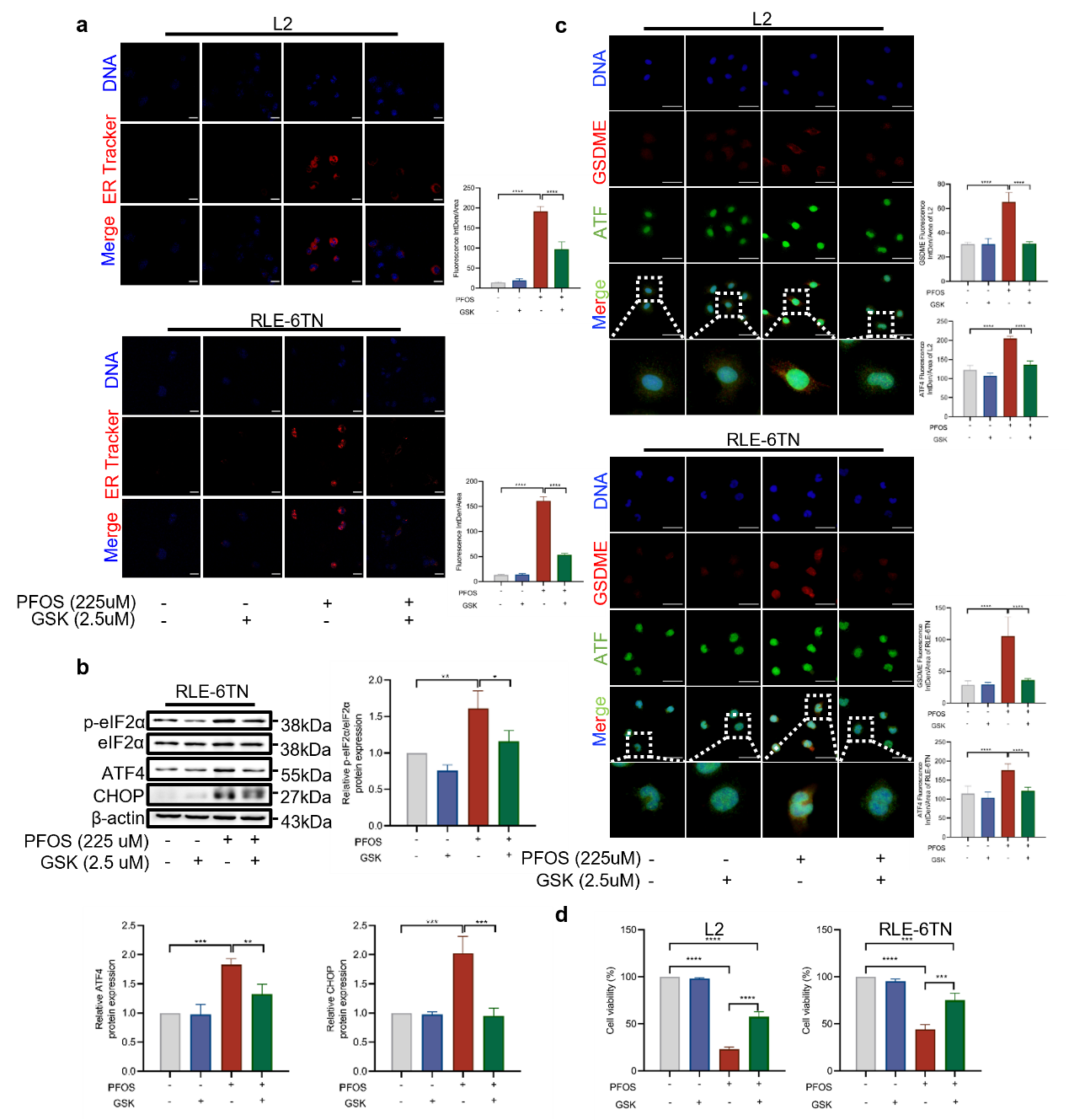


**Supplementary Fig. 6** Involvement of PERK signaling in PFOS effects on AECII. Cells were incubated with GSK (2.5μM) and/or PFOS (225μM) for 24 h. **a** Swollen ER levels are evaluated by ER staining (red). Scale bar, 20 μm. **b** Western blot analyses of p-eIF2α, ATF4 and CHOP. **c** The expression of GSDME (red), ATF4 (green) and DAPI (blue) in cells treated with or without GSK and/or PFOS for 24h in vitro. Scale bars, 20 μm. **d** Cell viability was measured after treated with or without GSK and/or PFOS for 24h. **d** The LDH release of AECⅡ. Scale bars, 100 μm. The data were presented as mean±SD of three replicates (n=3) with statistical significances of * *p*<0.05, ** *p*<0.01, *** *p*<0.001, or **** *p*<0.0001, respectively.


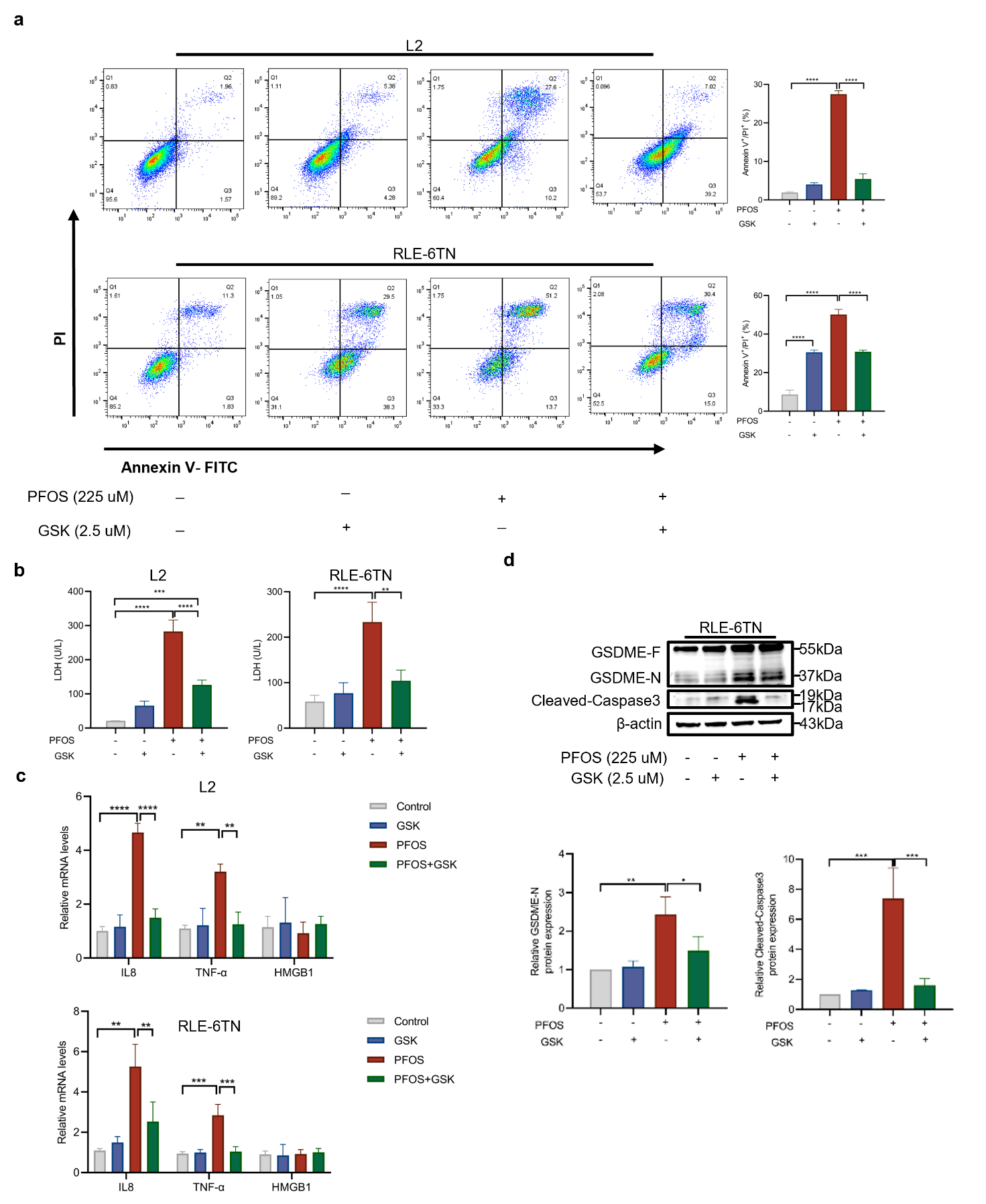


**Supplementary Fig. 7** Involvement of PERK/ATF4 signaling in GSDME-dependent AECⅡ pyroptosis induced by PFOS. Cells were incubated with GSK (2.5μM) and/or PFOS (225μM) for 24 h. **a** Flow cytometric determination of Annexin V+/PI+ AECIIs. **b** LDH was assayed in the medium of L2 and RLE-6TN cells. **c** mRNA levels of proinflammatory factor IL8, TNF-α, and HMGB1 in L2 and RLE-6TN cells. **d** Western blot analyses of cleaved-PARP, GSDME-N or cleaved-caspase-3 in L2 and RLE-6TN cells. The data were presented as mean±SD of three replicates (n=3) with statistical significances of * *p*<0.05, ** *p*<0.01, *** *p*<0.001, or **** *p*<0.0001, respectively.

**Supplementary Table 1**

| **Antigen** | **CATALOG** | **Dilutions** | **Manufacture** |
| --- | --- | --- | --- |
| DFNA5/GSDME | ab215191 | 1:1000 | Abcam |
| DFNA5/GSDME | EM1901-48 | 1:50 | HUABIO |
| Cleaved caspase-3 | #9664 | 1:1000 | Cell Signaling Technology |
| PARP | 66520-1-Ig | 1:20000 | Proteintech |
| p-PERK | #3179 | 1:1000 | Cell Signaling Technology |
| PERK | #3192 | 1:1000 | Cell Signaling Technology |
| p-eIF2α | #3398 | 1:1000 | Cell Signaling Technology |
| eIF2α | #5324 | 1:1000 | Cell Signaling Technology |
| CHOP | #2895 | 1:1000 | Cell Signaling Technology |
| ATF4 | EM1612-37 | 1:2000 | HUABIO |
| ABCA3 | ab24751 | 1:200 | Abcam |
| β-actin | #4967 | 1:1000 | Cell Signaling Technology |
| HRP-labeled goat  anti-rabbit IgG(H+L), | A0208 | 1:1000 | Beyotime |
| HRP-labeled goat  anti-mouse IgG(H+L) | A0216 | 1:1000 | Beyotime |
| goat anti-mouse IgG H&L  (Alexa Fluor® 594) | ab24751 | 1:400 | Abcam |
| goat anti-rabbit IgG H&L | ab24751 | 1:500 | Abcam |
